# Editorial: Modern achalasia: diagnosis, classification, and treatment

**DOI:** 10.3389/fmed.2024.1427398

**Published:** 2024-06-20

**Authors:** Flavio R. Takeda, Fernando A. M. Herbella, Leonardo M. Del Grande

**Affiliations:** ^1^Santa Casa Medical School, São Paulo, Brazil; ^2^Department of Surgery, Escola Paulista de Medicina, Federal University of São Paulo, São Paulo, Brazil

**Keywords:** esophageal, achalasia, POEM, myotomy, fundoplication

Achalasia is certainly the most understood primary esophageal motility disorder (1). However, especially in South America, it has been considered a differential diagnosis due to gastroesophageal reflux diseases and a benign esophageal disease leading to progressive dysphagia, regurgitation, heartburn, cough, and weight loss. In this Research Topic, we discussed all issues related to diagnosis, classification, and the treatment modalities for controlling the symptoms. The standard diagnosis of this pathology is made according to manometry, x-ray esophagogram, and clinical symptoms. Classification with high-resolution manometry and Chicago's classification can lead to distinct treatment. Nevertheless, several aspects of diagnosis and treatment are still elusive. In addition, a relatively novel therapy—peroral endoscopic myotomy (POEM) (2)—has become widespread and the manometric classification, in constant evolution, has acknowledged the possibility of variants other than the classic picture of aperistalsis and lack of relaxation of the lower esophageal sphincter (3).

Achalasia is very prevalent in South America due to the esophagopathy related to Chagas' Disease allowing surgeons and gastroenterologists to gain valuable experience with a high number of cases treated (4). A team of South American surgeons led this Research Topic series on achalasia. Four very interesting articles make up this series. A critical review performed by Tustumi evaluating non-conventional treatments for achalasia brings a discussion about some evidence of alternative surgical options such as cardiectomy, cardioplasty, or even esophagectomy, pharmacological therapy, local injections by endoscopy, and endoscopic devices. This article could also be of interest to patients who have issues with clinical conditions, frailty, or multiple surgical interventions with severe disabilities. Since POEM emerged as a minimally invasive procedure option for achalasia, the popularity of this procedure has risen quickly. Nabi and Reddy discussed the different techniques (location, thickness, and length of the myotomy) related to the relief of symptoms and post-procedure gastroesophageal reflux disease. The incidence of GERD after POEM is high. However, some publications on the endpoint of endoscopic erosive esophagitis and improved symptom relief (Eckardt score) are debatable. Olvera-Prado et al. discussed the persistent symptoms and chest pain that occur after laparoscopic myotomy and fundoplication of achalasia, showing that, besides the anatomical changes and improvement of esophageal emptying, some symptoms could last even if post-operative exams showed promising results.

Despite the negligible number of achalasia cases per year, its diagnosis and treatment have improved in recent years. The hot topic discussion remains related to POEM's efficacy and post-procedure control of dysphagia and GERD. Surgical procedures such as laparoscopic myotomy and partial fundoplication seem to be good choices; minimally invasive endoscopic procedures also have use as they attempt to control achalasia symptoms. We believe the whole series is worth reading.

## Author contributions

FT: Data curation, Writing – review & editing, Writing – original draft, Formal analysis. FH: Writing – review & editing, Writing – original draft, Visualization, Validation, Supervision, Software, Resources, Project administration, Methodology, Investigation, Funding acquisition, Formal analysis, Data curation, Conceptualization. LD: Writing – review & editing, Writing – original draft, Methodology, Conceptualization.
